# New Acetylcholinesterase Inhibitors for Alzheimer's Disease

**DOI:** 10.1155/2012/728983

**Published:** 2011-12-15

**Authors:** Mona Mehta, Abdu Adem, Marwan Sabbagh

**Affiliations:** ^1^The Cleo Roberts Center for Clinical Research, Banner Sun Health Research Institute, 10515 W Santa Fe Dr, Sun City, AZ 85351, USA; ^2^Department of Pharmacology and Therapeutics, United Arab Emirates University, Al Ain, UAE

## Abstract

Acetylcholinesterase (AChE) remains a highly viable target for the symptomatic improvement in Alzheimer's disease (AD) because cholinergic deficit is a consistent and early finding in AD. The treatment approach of inhibiting peripheral AchE for myasthenia gravis had effectively proven that AchE inhibition was a reachable therapeutic target. Subsequently tacrine, donepezil, rivastigmine, and galantamine were developed and approved for the symptomatic treatment of AD. Since then, multiple cholinesterase inhibitors (ChEI) continue to be developed. These include newer ChEIs, naturally derived ChEIs, hybrids, and synthetic analogues. In this paper, we summarize the different types of ChEIs in development and their respective mechanisms of actions. This pharmacological approach continues to be active with many promising compounds.

## 1. Introduction

Acetylcholinesterase (AChE) has proven to be the most viable therapeutic target for symptomatic improvement in Alzheimer's disease (AD) because cholinergic deficit is a consistent and early finding in AD. Inhibition of AChE was considered to be achievable as a therapeutic target because of proven efficacy of inhibition of peripheral AChE as a treatment for myasthenia gravis (MG) proving that the approach was feasible. However, selective inhibition of the central nervous system (CNS) AChE initially proved to be daunting. Before tacrine, physostigmine, the classic AChE inhibitor (AChEI) was investigated as a treatment for AD. Physostigmine was subsequently abandoned because of poor tolerability. Four drugs are currently available for AD treatment: galantamine, rivastigmine, donepezil, and memantine. The first three are AChE inhibitors and memantine is not.

There are two types of cholinesterase, AChE and butyrylcholinesterase (BuChE). AChE is found primarily in the blood and neural synapses. BuChE is found primarily in the liver. The biggest difference between the two is the substrates. AChE hydrolyzes acetylcholine (ACh) more quickly, and BuChE hydrolyzes butyrylcholine (BuCh) more quickly. BuCh is a synthetic compound used to distinguish AChE receptors from BuChE receptors. Many of the drugs that are available for treatment of AD target both AChE and BuChE, but some are more selective than others. In this paper, we will be focusing on older acetyl cholinesterase inhibitors (ChEIs), current ChEI, naturally derived ChEI, hybrid ChEI, and synthetic analogues.

## 2. Older Cholinesterase Inhibitors

### 2.1. Physostigmine (Eserine)

Physostigmine was the first ChEI investigated for the treatment of AD. It is isolated from the seeds of *Physostigma venenosum* as a parasympathomimetic plant alkaloid. Although it is able to pass through the blood-brain barrier (BBB), it has a short half-life and a narrow therapeutic index. It also has many side effects such as nausea, vomiting, headaches, diarrhea, and dizziness. Physostigmine was in use for MG, glaucoma, and delayed gastric emptying. However, the drug was not approved and was abandoned for AD use due to the disadvantages mentioned above. The newer drugs proved to be more effective with a lower side effect profile [1].

### 2.2. Tacrine

Tacrine was the first drug approved for treatment of AD in 1993 [2]. It is a potent inhibitor of both AChE and BuChE. Tacrine was approved both because of efficacy on the ADAS-Cog and on the global measure compared to placebo in phase II and phase III clinical trials of AD subjects [3]. However, widespread use of tacrine was limited as it was poorly tolerated as it caused a number of side effects including nausea, vomiting, dizziness, diarrhea, seizures, and syncope. Also, administration and compliance were challenging due to four times a day dosing regimen because of a short half-life. In addition, patients who used the drug required periodic blood monitoring due to hepatotoxicity [4]. Eventually, tacrine was discontinued due to the aforementioned liver toxicity which was thought to be caused by the affinity for BuChE [2] and because less toxic, better tolerated drugs with easier dosing schedule were approved.

### 2.3. Donepezil

Donepezil was approved in 1996 for the treatment of mild-to-moderate AD. A twelve-week double-blind study was performed by Rogers et al. A total of 468 AD patients were separated into three groups: placebo, low dose (5 mg/day), and high dose (5 mg/day for week 1 and then 10 mg/day thereafter). Improvements were seen as soon as three weeks, and clinically significant effects were seen at nine weeks. The side effects were comparable with the placebo for the most part. Patients who were on the high dose occasionally experienced transient nausea, diarrhea, and insomnia [5]. Donepezil is also thought to have an additional mechanism of action other than just as a ChEI. It is believed that donepezil does not act only at just the neurotransmitter level but also at a molecular and cellular level in nearly every stage involved with the pathogenesis of AD. These include, but are not limited to, inducing a neuroprotective isoform of AChE, blocking various aspects of the excitotoxic cascade induced by glutamate, mitigating the effects of oxidative stress, and reducing the expression of inflammatory cytokines [6]. Donepezil is approved for use in mild, moderate, and severe AD but not for other forms of dementia. It has shown some benefit in mild cognitive impairment [7, 8] but is not approved for this indication. Recently, a higher dose of 23 mg formulation was approved for use in moderate-to-severe AD subjects. In the US, generic donepezil is now available. Donepezil is well absorbed with a relative oral bioavailability of 100% and reaches peak plasma concentrations in 3 to 4 hours. The elimination half-life of donepezil is about 70 hours and is approximately 96% bound to human plasma proteins. Donepezil is metabolized by CYP 450 isoenzymes 2D6 and 3A4 and undergoes glucuronidation.

### 2.4. Rivastigmine

Rivastigmine is a small molecule and has easy BBB permeability. Rivastigmine also has BuChE and AChE inhibitory properties. Rivastigmine was approved for the treatment of mild-to-moderate AD in 2000 and has since gained approval for Parkinson's related dementias. Corey-Bloom et al. conducted a trial with 699 patients with AD ranging from mild-to-moderately severe. The patients were split into three groups: a placebo group, a group on 1–4 mg/day, and a group on 6–12 mg/day for 26 weeks. Patients in the 6–12 mg/day group demonstrated significant improvements in cognition (ADAS-cog 4.94 points), activities of daily living, global assessment of change, and the Mini-Mental State Examination (MMSE) [9]. In capsular form, rivastigmine has been frequently associated with side effects of nausea, vomiting, anorexia, and diarrhea. In 2007, rivastigmine was reformulated for delivery through a transdermal patch. This has resulted in significantly lower GI side effects compared to the oral capsule. It is suitable to be converted to a patch delivery system because it is a small molecule (<300 Da in size), and it is both lipophilic and hydrophilic. It is minimally metabolized by CYP450 cytochrome system with weak binding to plasma proteins (approximately 40%). The duration of anticholinesterase activity in cerebrospinal fluid is approximately 10 hours after a 6-mg oral dose.

### 2.5. Galantamine

The therapeutic action of galantamine has been reported to be mainly produced by its sensitizing action on nAChRs rather than by general cholinergic enhancement due to cholinesterase inhibition. Galantamine was approved for the treatment of mild-to-moderate AD in February 2001. The trial conducted by Tariot et al. in 2000 consisted of 978 patients randomized to galantamine or matching placebo for five months. Administration of galantamine was associated with a significant improvement on the ADAS-cog score with 3.3 points for the 16 mg/day group and 3.6 points for the group on 24 mg/day when compared to placebo. Discontinuation of treatment due to adverse effects was minimal in both groups and equal to the placebo group. Both of the treatment groups showed significant improvement in the behavioral, cognitive, and functional symptoms of AD. Having a slow escalation of the dosage increased the tolerability of the drug [10]. Absorption of galantamine is rapid and complete and shows linear pharmacokinetics. It is well absorbed with absolute oral bioavailability between 80 and 100%. It has a half-life of seven hours. Peak effect of inhibiting acetylcholinesterase was achieved about one hour after a single oral dose of 8 mg in some healthy volunteers. Plasma protein binding of galantamine is about 18%.

### 2.6. Metrifonate

Metrifonate is a long-acting irreversible ChEI that was originally used to treat schistosomiasis. Although there was a low risk of side effects with short-term use, long-term use caused respiratory paralysis and neuromuscular transmission dysfunction similar to a myasthenic crisis. As such, the FDA submission was halted and the clinical trials were discontinued during phase III. However, it did demonstrate a robust therapeutic effect on ADAS-cog and other measures. But, until the relationship between metrifonate and neuromuscular dysfunction is further explored, metrifonate is not an option for AD treatment at this time [11]. Metrifonate is not an approved AD treatment but shows efficacy that was outweighed by safety risks.

### 2.7. Older ChEIs Summary

Dozens of short-term clinical trials, open-label extension studies, and long-term clinical cohort observational studies generally point to the following (a) current ChEIs (donepezil, rivastigmine, and galantamine) reduce cognitive, functional, and behavioral decline in AD, (b) their efficacies appear similar (there is no level 1 grade data that shows otherwise), (c) their benefits are sustained with treatment persistence, (d) their benefits are generally dose-related (until limited by side-effects at very high doses), and (e) they appear to be relatively safe and well-tolerated (see Table 1).

## 3. New Cholinesterase Inhibitors

### 3.1. Physostigmine Derivatives

#### 3.1.1. Phenserine

Phenserine is the phenylcarbamate derivation of physostigmine that has a dual effect: decreasing beta-amyloid precursor protein and has a reversible AChE inhibition. It has a quick absorption rate and is less toxic than physostigmine and tacrine. It has also shown improved learning and memory in aging rats and dogs. Another study involving dogs showed that phenserine improved learning and memory compared to dogs receiving placebo group [1]. Phase III clinical trials were conducted during 2003-2004 in which 384 AD patients recruited. The trials were conducted using 10 mg and 15 mg twice daily doses. However, phenserine did not achieve significant improvement on the ADAS-cog scores compared to placebo. Subsequently, further clinical trials for AD have been abandoned [1]. Post hoc analysis of all three Phase III clinical trials identified that the group of subjects receiving the highest tested dose (15 mg per day) for more than 12 weeks demonstrated a statistically significant benefit of phenserine over placebo in ADAS-cog, but only a trend toward improvement in the CIBIC+ measure.

#### 3.1.2. Tolserine

Tolserine only differs from phenserine at the 2′-methyl substitution on its phenylcarbamoyl moiety. Preclinical studies were initiated in 2000, and it was shown to be 200-fold more selective against hAChE versus BuChE [1]. Tolserine proved to be a highly potent inhibitor of human AChE compared to its structural analogues physostigmine and phenserine. However, there are no published reports on Tolserine in humans so it is difficult to identify potential risks and benefits in preclinical or clinical models.

#### 3.1.3. Esolerine

Zhan et al. conducted a study in which a series of physostigmine analogues were prepared and assessed for ChEI. Compound 17, a cyclic alkyl carbamate of esolerine, a very strong AChE inhibitor and significantly favored AChE over BuChE [12]. Esolerine is a tacrine derivative, but there are no published reports in humans so it is difficult to identify potential risks and benefits in preclinical and clinical models.

### 3.2. NS2330 (Tesofensine)

Both *in vitro* and *in vivo* studies have shown that tesofensine inhibits the presynaptic uptake of the neurotransmitters serotonin, norepinephrine, and dopamine. The compound enhances the function of the neurotransmitters acetylcholine, noradrenaline, and dopamine, which are all impaired in Alzheimer's patients. A decrease in beta-amyloid concentration was found in mice after use of tesofensine which was thought to be a neuroprotective effect. Phase IIA trials showed significant cognitive improvement in those with mild AD. However, Phase IIB trials showed limited activity. The trials were discontinued in 2008. Currently, tesofensine is being marketed as a drug against obesity [13, 14].

## 4. Naturally Derived

### 4.1. Huperzine A

Huperzine A (HupA) is a Lycopodium alkaloid isolated from the Chinese medicinal herb *Huperzia serrata* used for memory deficiency. *Huperzia serrata* is widely grown in China and Chinese medical tradition emphasize herbal remedies. It has attracted much attention because it is a highly selective, reversible, and potent AChE inhibitor [15]. The synthetic racemic mixture of HupA has less AChE inhibitory effects than the natural kind. Many of the initial derivates demonstrated lower potency than the natural HupA. HupA has also been hybridized with tacrine and donepezil. While the donepezil hybrid proved to be less effective, the HupA-tacrine hybrids called huprines Y and X have shown to be more effective in amplifying AChE *in vitro* than tacrine. Huprines Y and Z were shown to have better inhibitory activity than either of the parent drugs as well [1]. HupA has a higher oral bioavailability compared to tacrine and donepezil. The improvement is more noticeable on working memory than reference memory as compared to tacrine and donepezil as well [16]. HupA is also shown to be more potent than tacrine, rivastigmine, and galantamine in terms of inhibition activities, and it had the least amount of activity against BuChE [15].

HupA is considered to be the drug of choice in China for the treatment of memory disorders. It is also in Phase II clinical trials in Europe [16]. Phase II trials have also been conducted in over 30 sites in the US. A trial using a lower dose of 200 mcg twice daily showed no improvement on the ADAS-cog scores, but a different trial using a higher dose of 400mcg twice daily showed statistically significant improvement in the ADAS-cog scores compared to placebo [15, 17] with GI side effects reported. Since it is marketed in the US as a nutraceutical supplement because it lacks a proprietary patent for the treatment of AD, FDA approval is not being pursued. It is widely available as a supplement marketed to enhance memory but with no labeling to treat specific diseases.

### 4.2. Huperzine B

Natural Huperzine B (HupB) is a Lycopodium alkaloid isolated from the Chinese medicinal herb *Huperzia serrata* which has been demonstrated as an effective and reversible inhibitor of AChE. HupB is less potent and selective than HupA, but it has higher therapeutic index and other positive benefits. HupB derivatives were created to be more potent than natural HupB. A novel series of 16-substituted derivatives were synthesized. 9i has the highest potency *in vitro*. The efficacy of 9i was more potent *in vitro* than HupA, galantamine, and rivastigmine, and it was equivalent to donepezil. 9c and 9i showed moderate neuroprotection against H_2_O_2_-induced neurotoxicity [18]. However, given the ubiquitous availability of HupA and its higher potency, clinical development of HupB has been reportedly limited. Side effects would be expected to be similar in nature (GI) to HupA and other ChEIs.

### 4.3. *Nelumbo nucifera*

*Nelumbo nucifera* is an aquatic plant with numerous medicinal properties. It has recently demonstrated that the stamens fed to rats performing maze learning tasks improved memory. The MOA is felt to be AChE inhibition. One new compound and four known compounds were isolated from the n-butanol fraction of the *N. nucifera* for the first time. The new compound is a beta-cyclogeraniol diglycoside, nuciferoside (**5**), and the four known compounds are cycloartenol (**1**), *p*-hydroxybenzoic acid (**2**), vanilloloside (**3**), and 5′-*O-*methyladenosine (**4**). Compounds **5** and **1**–**3** demonstrated good and noncompetitive AChE inhibition and compounds **1, 2, **and **5** showed exhibited BuChE inhibition. Compounds **1**–**3** and **5** have possible ChE inhibitory effects with the potential to be used for AD treatment. The primary effect would be as an AChE inhibitor rather than as BACE1 inhibitors [19]. There are no reports of human studies. Preclinical and clinical safety and toxicity data are not reported.

### 4.4. *Himatanthus lancifolius*

*Himatanthus lancifolius* is a shrub that contains several indole alkaloids with a number of medicinal properties such as antimicrobial effects, gastroprotection, and the ability to affect the vascular and nonvascular smooth muscle responsiveness. Seidl et al. [20] conducted a study to determine if there were any AChE inhibiting properties from the *Himatanthus lancifolius *extract and uliene *in vitro*. The dichloromethane (DCM), and ethyl acetate (EtOAc), fractions showed significant AChE inhibitory effects. Uliene was the significant compound present in both fractions [20]. There are no reports of human studies specific to cognition. Preclinical and clinical safety and toxicity data are not reported.

### 4.5. Galangin

Guo et al. studied 21 different flavonoids for potential AChE inhibition properties in the brain *in vitro*. Flavonoids have been of great interest in AD research and treatment because of their free radical scavenging properties. Epidemiological evidence suggests that higher consumption of flavonoids is associated with lower incidences of AD. A flavonol isolated from Rhizoma Alpiniae Officinarum called galangin demonstrated the highest inhibitory effects on AChE activity. However, it is unknown if the galangin binds to the AChE directly or has the same binding site as the ATCh substrate [21]. Nevertheless, this suggests that galangin could be developed as a potential treatment for AD because of the dual MOA of ChEI and free radical scavenging properties. There are no human studies reported to date. Clinical and preclinical toxicities have not been established.

### 4.6. Cardanol Derivatives

de Paula et al. designed new AChEI from nonisoprenoid phenolic lipids (NIPLs) of *Anacardium occidentale*. Cardols, cardanols, anacardic acids, and methylcardols are the primary NIPL components of cashew nut-shell liquid (CNSL) and have been used to generate potential bioactive compounds. The derivates have structural, electrical, and hydrophobic properties that are relevant to recognition of AChE molecules. The study concluded that the most promising candidates to the development of AChEI for AD treatment were derived from cardanol [22]. Development of cardanol is appealing because of the abundancy of the raw material source. There are no human studies reported to date. Clinical and preclinical toxicities have not been established.

## 5. Hybrids

Since AD is a multifactorial disease, the innovative model is of the “one molecule, multiple targets” approach. Hybrids combine BBB permeability with drugs targeting multiple receptors or epitopes. Further, hybrids offer the promise of dual MOAs and added potency. The multipotent approach includes novel tacrine-melatonin hybrids, dual inhibitors of AChE and MAO or serotonin transporters, potent cholinesterase inhibitors with antioxidant and neuroprotective properties, gallamine-tacrine hybrids binding at cholinesterases and M_2_ muscarinic receptors, NO-donor, tacrine hybrids as hepatoprotective drugs focusing on AD or fluorescent tacrine, coumarin hybrids [23]. The side effects profiles are unknown in humans at present.

### 5.1. 5-(N-Methyl-N-propargyl-aminomethyl) Quinolin-8-yl Dimethyl Carbamate (2) and 5-(N-Methyl-N-propargylaminomethyl) Quinolin-8-yl Ethylmethyl Carbamate (3)

One type of new drug is a site-activated chelator that inhibits both AChE and MAO A/B. Although the original chelators (clioquinol and desferrioxamine) had success in clinical trials, the drugs did not selectively target the biometals in the brain. Therefore, the therapeutic use was limited due to toxicity (in the case of clioquinol) or poor selectivity and permeability. Now, chelators are designed with MAO A/B inhibitory activity to enhance efficacy and as prodrugs to enhance targeting. This new prochelator would act as HLA 20A in that it would selectively inhibit AChE with minimal metal ion binding affinity, possess increased activity against MAO A/B, and it would be activated by AChE, which is located primarily in the brain, to release the active chelator M30. The two compounds synthesized are compounds 2 and 3. Compounds 2 and 3 are like rivastigmine in that the AChE inhibited progressively increases as incubation time increases. Compound 2 is more potent and 3 is less potent than rivastigmine in blocking AChE at 1 *μ*M. Both compounds are more potent at inhibiting MAO-A than M30, but compound 2 is less potent than M30 at inhibiting MAO-B and compound 3 has minimal inhibitory effects. The biggest novelty is that M30 is combined with these compounds into prochelators. Compound 2 is more promising of the two [24].

### 5.2. Donepezil and AP2238

AP2238 is the first published compound to bind both anionic sites of AChE. The potency against AChE is comparable to donepezil, while its ability to contrast beta-amyloid aggregation is higher. Rizzo et al. reports on a series of hybrids developed from donepezil and AP2238 in which the idanone core from donepezil is linked to the phenyl-N-methylbenzylamino moiety from AP2238. A derivate in which the phenyl-N-methylbenzylamino moiety from AP2238 was replaced by phenyl-N-ethylbenzylamino moiety from AP2243 and the idanone ring was replaced by a tetralone scaffold. A total of 22 compounds were synthesized. Derivates 21 and 22 were the most active of all the compounds, and the potency was equal to the reference compounds. Compounds 15, 21, and 22 had a 5-carbon alkyl chain with an amino moiety at one end which gave the compounds better contact at the peripheral anionic site (PAS). This improved the inhibition of AChE-induced aggregation vastly when compared to the reference compounds. Compound 21 was a part of the tetralone series, and the methoxy substituent in position 6 was replaced by a pentyl chain. Compound 22 was a piperidine derivative of the tetralone series. In compound 15, the second amino moiety was replaced by piperidine with *n* = 5. Overall, compounds 15, 21, and 22 showed the most promise [25]. There are no reports of human studies. Preclinical and clinical safety and toxicity have not been established.

### 5.3. Donepezil-Tacrine Hybrids

Camps et al. designed a novel series of donepezil-tacrine hybrids, which interact simultaneously at the peripheral, active, and midgorge binding sites of AChE. They are desirable because both compounds have known efficacy and BBB permeability with slightly different MOAs. They were tested for the ability to inhibit BuChE, AChE, and beta-amyloid aggregation induced by AChE. The compounds are combination of the 5,6-dimethoxy-2-[(4-piperidinyl)methyl]-1-idanone moiety of donepezil and 6-chlorotacrine. All of the new hybrids have proven to be highly potent hAChE inhibitors. All of the new compounds also demonstrated significant inhibition of beta-amyloid aggregation and were shown to be more potent than parent compounds [26].

### 5.4. Tacrine/Ferulic Acid Hybrids and Beta-Carboline Derivatives

Three beta-carboline derivatives (referred to as without chemical names (2A, 2B, 2C)) and two tacrine/ferulic acid hybrids (1A, 1B) were tested *in vivo* in three-month-old female rats. The tacrine/ferulic acid hybrids showed higher AChE inhibition and comparable BuChE inhibition when compared to tacrine *in vitro*. However, neither compounds 1A nor 1B showed any therapeutic effects against scopolamine-induced cognitive deficits *in vivo*. Compound 1B actually worsened the impairment. Compound 2C was shown to be the most effective of the three *in vitro*. Compound 2B did not show any significant activity, and compound 2A was comparable to galantamine. However, none of these three compounds were shown to be effective *in vivo*. Despite the promising *in vitro *information, none of the five compounds are suitable for AChE inhibition against AD [27].

### 5.5. Tacrine-8-hydroxyquinoline Hybrids

Both tacrine and PBT2 (an 8-hydroxyquinoline derivative) are known for ChE inhibition and decreasing beta-amyloid concentrations, respectively. Fernandez-Bachiller et al. designed, synthesized, and evaluated novel tacrine-8-hdroxyquinoline hybrids as possible therapeutic drugs for AD treatment. The hybrids were found to be more effective than tacrine against AChE and BuChE. They also show low cell level toxicity and could possibly penetrate the CNS as demonstrated by an *in vitro* BBB. The drug has also demonstrated antioxidant and copper-complexing properties. With all the properties that these hybrids possess, it could be a possible therapeutic drug treatment *in vivo* as well [3].

### 5.6. Other Hybrids Combinations

Samadi et al. studied tacripyrines and developed a new compound called p-methoxytacripyrine (RL2/101). RL2/101 impedes the proaggregation beta-amyloid effect of hAChE and has mild inhibitory effects on the self-aggregation of A*β* 42. RL2/101 is a potent Ca2+ antagonist that passes through the blood-brain barrier (BBB). It also displays antioxidant and neuroprotective properties. Samadi et al. carried out with the synthesis of a number of series of simple, and readily available 2-aminopyridine-3,5-dicarbonitriles (3–22), and 2-chloropyridine-3,5-dicarbonitriles (21–28) derived from 2-amino-6-chloropyridine-3,5-dicarbonitrile (1) and 2-amino-6-chloro-4-phenylpyridine-3,5-dicarbonitrile (2). Compounds 3, 4, 21–23, 25, 26 were shown to be highly selective for hAChE. Compounds bearing small groups such as the N, N^0^-dimethylamino or the pyrrolidino preferentially inhibit AChE. Compounds 3, 17, 22, 24, and 26 showed the highest neuroprotection values. These compounds could be further developed to have multifaceted approach against cholinergic dysfunction and oxidative stress [23].

Camps et al. designed two new series of hybrid AChEIs and tested them for AChE inhibition, butyrylcholinesterase, BACE-1, and self-induced and AChE-induced beta-amyloid aggregation and BBB permeability. The hybrids are composed of a 6-unit chlorotacrine and a multicomponent reaction-derived pyrano [3,2-c]-quinoline scaffold as the active and peripheral sites interacting moieties, respectively. Due to the dual binding site activity, both hybrids demonstrate the ability to inhibit AChE-induced beta-amyloid_1-40_ aggregation and self-induced beta-amyloid_1-42_ aggregation. Some of compounds can also inhibit BACE-1. These hybrids also have the ability to permeate the BBB. Hybrid 27 emerged as the most promising therapeutic as it was able to target both AChE and beta-amyloid production and aggregation [28].

## 6. Synthetic Analogues

### 6.1. Phenyl-5,6-dimethoxy-1-oxo-2,3-dihydro-1H-2-indenylmethanone Analogues

Synthetic analogues are under development because, with targeted pharmacological development, class-specific hepatotoxicity and known gastrointestinal side effects may be avoided. The risk of developing synthetic analogs is that they might not have the potency and BBB permeability as naturally derived ChEIs or they may have unanticipated pharmacological properties. Ali et al. synthesized phenyl-5,6-dimethoxy-1-oxo-2,3-dihydro-1H-2-indenylmethanone analogues labeled **5a–5n**. Their data showed that the majority of the compounds had moderate AChE inhibitory effects. **5f** had the most significant effects followed by **5e** and then **5a**. The study suggests that having an OCH_3_ group substituted phenyl ring at diketone derivatives creates a vast improvement in AChE inhibition [29].

### 6.2. N-Alkyl-7-methoxytacrine Hydrochlorides

The first AChEI developed was THA, but using the drug caused dose-dependent but reversible liver toxicity. 7-methoxytacrine (7-MEOTA) is an analogue of THA that has far less toxicity and is pharmacologically equivalent to THA. In a study by Korabecny, fourteen 7-MEOTA analogues were synthesized. Models of human recombinant AChE (hAChE) and human plasmatic BuChE (hBuChE) were used to evaluate these new analogues *in vitro* and were compared to THA and 7-MEOTA. Compounds 5–7 showed better inhibition of hAChE than compared to 7-MEOTA and THA, especially compound 5 as it was found to be five times more potent than THA. Compounds 9, 10, and 14 were found to be ineffective against hAChE. The structure-activity relationship findings point at the C6-C7 N-alkyl chains for cholinesterase inhibition [30].

### 6.3. Ladostigil

Ladostigil is a novel anti-Alzheimer's disease drug, with neuroprotective, multimodal brain-selective monoamine oxidase, and cholinesterase inhibitor properties [31]. It has neuroprotective and antioxidant activities in cellular models at much lower concentrations than those inhibiting MAO or AChE. When ladostigil (1 mg/kg/day) was given for 6 months to 16-month-old rats, it prevented the age-related increase in activated astrocytes and microglia in several hippocampal and white matter regions and increased proNGF immunoreactivity in the hippocampus towards the levels in young rats.

Ladostigil also prevented the age-related reduction in cortical AChE activity and the increase in butyrylcholinesterase activity in the hippocampus, in association with the reduction in gliosis. The immunological and enzymatic changes in aged rats were associated with improved spatial memory. Ladostigil treatment had no effect on memory, glial, or proNGF immunoreactivity in young rats [32]. Ladostigil ((N-propargyl-(3R) aminoindan-5yl)-ethyl methyl carbamate) is, presently in a Phase IIb clinical trial and intended for the treatment of Alzheimer's disease and dementia comorbid with extrapyramidal disorders and depression [31].

## 7. Discussion

A great deal of research has been done regarding AChEI as a therapeutic drug for AD. All of the current first-line treatments in the US are AChEIs. Physostigmine was the first ChEI investigated for AD, but it was discarded due to a number of disadvantages. Although phenserine, a physostigmine derivative, has failed the Phase II clinical trials, there are other derivatives in development. In addition, metrifonate also has a profound effect on those with AD. A metrifonate derivative with the same or better efficacy and a lesser side effect profile would be a promising therapeutic drug. Naturally derived AChEIs are also a promising area of interest. HupA is the drug of choice in China [16]. Many hybrids are also being created. Some hybrids have completely new materials, but other hybrids are using the older AChEI and are trying to improve upon them. The synthetic analogues have only been tested *in vitro* at this time, but it is an area that can help direct future treatment options for AD. Although the majority of the drugs discussed in this paper have not yet been tested in animals or humans, each of these areas will continue to develop because this class of drugs has demonstrated its value in symptomatic therapy. This class of drugs will continue to be developed because it is a proven symptomatic therapy with a recognized target. Drugs in the class have a proven track of CNS permeability, known side effect profile, and demonstrated efficacy. It is logical to consider further development of novel agents in this class. Challenges to development include potency, safety, and side effects as well as comparison to current ChEIs, many of which are generic. Many compounds developed to date have no human or animal data. Thus, safety, efficacy, and toxicity have not been established. Further, this class of medications has not been established to possess disease-modifying properties. The risk of developing newer ChEIs is that they will need to be more effective than donepezil, rivastigmine, and galantamine to garner approval since these three drugs are FDA approved. Future research in this class will need to focus on whether ChEIs directly affect the pathophysiology of AD.

## Figures and Tables

**Table 1:** Summary of ChEIs in preclinical and clinical development.

Drug	Disposition
Physostigmine	First drug investigated; however, it is no longer used due to side effects, short half-life, and better treatment options.
Donepezil	Highly selective AChEI. Approved for mild, moderate, and severe AD.
Rivastigmine	Has dual bChEI and AChEI properties. Approved for mild-to-moderate AD. Patch formulation has reduced cholinergic-related side effects.
Galantamine	A lower potency AChEI with allosteric nicotinic receptor modulation properties.
Metrifonate	A highly selective AChEI that demonstrated a robust and significant clinical effect, but was abandoned after Phase III RCTs because of risk of neuromuscular dysfunction.
Phenserine	A derivative of physostigmine with a dual mechanism of action including anti-A*β* properties as well as AChEI. Despite a good safety profile, it did not achieve significant efficacy during Phase II trials.
Tolserine	A physostigmine derivative, has shown promise in the preclinical stages.
Esolerine	Another physostigmine derivative, is also a strong AChEI and favors AChE over BuChE greatly. It has not entered clinical trials.
NS2330 (tesofensine)	Robust preclinical efficacy and safety data. Early Phase II studies showed a positive signal on cognition. Follow-up RCTs for AD were discontinued in 2008 because of lack of clinical efficacy signal. It is currently being investigated as a treatment for obesity treatment.
Huperzine A	HupA is natural herb that is marketed as a supplement in the US. It acts as a ChEI. It is drug of choice for AD in China. Phase II trials in the US showed a modest but clinical significant effect on cognition in AD.
Huperzine B	HupB is less potent and selective than HupA, but it has a higher therapeutic index.
*Nelumbo nucifera*	The stamens of *Nelumbo nucifera* has demonstrated an improvement in memory in rats and favors AChE over BuChE.
*Himatanthus lancifolius*	A shrub with multiple medicinal purposes. The uliene in *Himatanthus lancifolius* is what is likely responsible for the significant AChE inhibitory effects.
Galangin	Galangin is a flavonoid that demonstrated significant inhibition of AChE. It has not been tested in human trials.
Donepezil hybrids	(1) Of the series of hybrids derived from Donepezil and AP2238, compounds 15, 21, and 22 demonstrated the most potential. Human studies have not been undertaken yet.
	(2) The entire series of donepezil-tacrine hybrids showed more significant benefits than either parent drug alone. Human studies are planned.
Tacrine hybrids	(1) The beta-carboline derivatives (2A, 2B, 2C) and tacrine/ferulic acid hybrids (1A, 1B) were shown to have no efficacy *in vivo, *and 1B actually worsened the impairment in an scopolamine-induced *in vivo* model. Clinical development has not been pursued further.
	(2) The tacrine-8-hdroxyquinoline hybrids showed potential *in vitro*, but the effects have yet to be shown *in vivo*.
Synthetic analogues	(1) The majority of the phenyl-5,6-dimethoxy-1-oxo-2,3-dihydro-1H-2-indenylmethanone analogues demonstrated significant AChEI in *in vitro *and *in vivo models*.
	(2) N-alkyl-7-methoxytacrine hydrochlorides are also an area of interest as compounds 5–7 have more efficacy than THA and 7-MEOTA.
